# Comparative evaluation of microleakage and internal voids in class II restorations using open sandwich technique: An *in vitro* study

**DOI:** 10.6026/973206300223254

**Published:** 2026-06-30

**Authors:** Kirti Rathee, Reena Rani, Parthivi Singh, Heena Gandhi, Lakshika Sharma, Sagar Hiwale

**Affiliations:** 1 Department of Conservative Dentistry and Endodontics, Institute of Dental Studies & Technologies, Modinagar, Ghaziabad, Uttar Pradesh, India; 2 Department of Pedodontics and Preventive Dentistry, Santosh Dental College and Hospital, Ghaziabad, Uttar Pradesh, India; 3 Department of Conservative Dentistry and Endodontics, Peoples Dental Academy, Peoples University, Bhopal Madhya Pradesh, India; 4 Department of Conservative Dentistry and Endodontics, Kalinga Institute of Dental Sciences, Bhubaneswar, Odisha, India; 5 Department of Conservative Dentistry and Endodontics, ITS Dental College, Muradnagar, Ghaziabad, Uttar Pradesh, India; 6 Department of Conservative Dentistry and Endodontics, Inderprastha Dental College and Hospital, Ghaziabad, Uttar Pradesh, India

**Keywords:** Microleakage, internal voids, Class II restorations, sandwich technique, ACTIVA Bioactive, composite restoration

## Abstract

The achievement of a durable and hermetic seal at the tooth restoration interface remains a significant challenge in restorative dentistry, particularly with Class II composite restorations. Therefore, it is of interest to compare microleakage and internal voids in Class II restorations using different intermediate materials in the open sandwich technique. The materials evaluated included ACTIVA Bioactive, Resin-modified Glass Ionomer Cement (RMGIC), Smart Dentin Replacement (SDR) and a control group with nanohybrid composite. Data showed that ACTIVA Bioactive showed the least microleakage and void formation, followed by RMGIC and SDR. Thus, we show the superior performance of bioactive materials in improving marginal sealing and reducing void formation in composite restorations.

## Background:

In contemporary restorative dentistry, the emphasis has shifted from merely replacing lost tooth structure to achieving biologically compatible and aesthetically superior restorations [1]. Advances in adhesive systems and resin-based composite materials have significantly enhanced the predictability of restorative procedures. However, despite these developments, one of the most persistent clinical challenges remains the achievement of a durable and hermetic seal at the tooth-restoration interface [2]. Polymerization shrinkage of resin composites results in volumetric contraction, which generates internal stresses and subsequently leads to marginal gap formation and microleakage. This phenomenon compromises restoration longevity by permitting bacterial ingress, post-operative sensitivity, marginal discoloration and secondary caries formation [3]. Bonding to enamel is relatively predictable due to its high inorganic content and uniform structure, whereas dentin presents a far more complex substrate. The heterogeneous composition of dentin, characterized by varying proportions of hydroxyapatite, collagen fibrils, dentinal tubules and intrinsic moisture, complicates the bonding process [4]. Additionally, cementum, with its lower mineral content and structural variability, poses further challenges in achieving reliable adhesion. Consequently, the gingival margins of Class II restorations, often located below the cementoenamel junction (CEJ), are particularly prone to microleakage [5]. Clinical placement of restoration margins in these subgingival regions exacerbates the difficulty due to limited accessibility, compromised moisture control and suboptimal finishing conditions, thereby necessitating enhanced strategies to ensure marginal integrity [6]. Another critical concern associated with Class II composite restorations is the formation of internal voids. These voids, which may arise during material placement or polymerization, can appear as radiolucent areas on radiographs and may be misdiagnosed as recurrent caries [7]. Furthermore, the presence of voids weakens the structural integrity of the restoration and facilitates microleakage by providing pathways for fluid and bacterial penetration. Despite advancements in composite formulations, the complete elimination of void formation remains elusive [8]. To mitigate the adverse effects of polymerization shrinkage and improve marginal adaptation, the sandwich or laminate technique was introduced, wherein an intermediate material is placed between the tooth substrate and the composite restoration [9]. This interfacial layer acts as a stress-absorbing buffer, reducing shrinkage-induced stresses and improving adaptation. Materials such as conventional glass ionomer cement (GIC), resin-modified glass ionomer cement (RMGIC) and flowable composites have been widely employed for this purpose [10]. While conventional GIC demonstrated limitations such as moisture sensitivity and material degradation over time, RMGIC offered improved mechanical properties, enhanced adhesion and reduced microleakage. However, its increased viscosity may hinder optimal adaptation [11]. Flowable composites, due to their superior adaptability and ease of placement, have been advocated as intermediate materials, although studies have reported inconsistent marginal sealing. More recently, Smart Dentin Replacement (SDR), a bulk-fill flowable composite with reduced polymerization stress and enhanced flow ability, has gained attention for its potential to improve internal adaptation and marginal integrity [12]. In addition, the emergence of bioactive restorative materials such as ACTIVA Bioactive represents a paradigm shift toward biomimetic dentistry. This material exhibits ion-releasing capacity, improved fracture resistance and potential self-sealing properties, although its effectiveness in preventing microleakage and void formation requires further validation [13]. Therefore, it is of interest to assess the microleakage and internal voids in Class II restorations using different intermediate materials with the open sandwich technique.

## Methodology:

In contemporary restorative dentistry, the emphasis has shifted from merely replacing lost tooth structure to achieving biologically compatible and aesthetically superior restorations. Advances in adhesive systems and resin-based composite materials have significantly enhanced the predictability of restorative procedures. However, despite these developments, one of the most persistent clinical challenges remains the achievement of a durable and hermetic seal at the tooth-restoration interface. Polymerization shrinkage of resin composites results in volumetric contraction, which generates internal stresses and subsequently leads to marginal gap formation and microleakage. This phenomenon compromises restoration longevity by permitting bacterial ingress, post-operative sensitivity, marginal discoloration and secondary caries formation. Bonding to enamel is relatively predictable due to its high inorganic content and uniform structure, whereas dentin presents a far more complex substrate. The heterogeneous composition of dentin, characterized by varying proportions of hydroxyapatite, collagen fibrils, dentinal tubules and intrinsic moisture, complicates the bonding process. Additionally, cementum, with its lower mineral content and structural variability, poses further challenges in achieving reliable adhesion. Consequently, the gingival margins of Class II restorations, often located below the cementoenamel junction (CEJ), are particularly prone to microleakage. Clinical placement of restoration margins in these subgingival regions exacerbates the difficulty due to limited accessibility, compromised moisture control and suboptimal finishing conditions, thereby necessitating enhanced strategies to ensure marginal integrity. Another critical concern associated with Class II composite restorations is the formation of internal voids. These voids, which may arise during material placement or polymerization, can appear as radiolucent areas on radiographs and may be misdiagnosed as recurrent caries. Furthermore, the presence of voids weakens the structural integrity of the restoration and facilitates microleakage by providing pathways for fluid and bacterial penetration. Despite advancements in composite formulations, the complete elimination of void formation remains elusive. To mitigate the adverse effects of polymerization shrinkage and improve marginal adaptation, the sandwich or laminate technique was introduced, wherein an intermediate material is placed between the tooth substrate and the composite restoration. This interfacial layer acts as a stress-absorbing buffer, reducing shrinkage-induced stresses and improving adaptation. Materials such as conventional glass ionomer cement (GIC), resin-modified glass ionomer cement (RMGIC) and flowable composites have been widely employed for this purpose. While conventional GIC demonstrated limitations such as moisture sensitivity and material degradation over time, RMGIC offered improved mechanical properties, enhanced adhesion and reduced microleakage. However, its increased viscosity may hinder optimal adaptation. Flowable composites, due to their superior adaptability and ease of placement, have been advocated as intermediate materials, although studies have reported inconsistent marginal sealing. More recently, Smart Dentin Replacement (SDR), a bulk-fill flowable composite with reduced polymerization stress and enhanced flow ability, has gained attention for its potential to improve internal adaptation and marginal integrity. In addition, the emergence of bioactive restorative materials such as ACTIVA Bioactive represents a paradigm shift toward biomimetic dentistry. This material exhibits ion-releasing capacity, improved fracture resistance and potential self-sealing properties, although its effectiveness in preventing microleakage and void formation requires further validation. Given the diversity of available intermediate materials and the limited comparative evidence regarding their performance, particularly in relation to margin location relative to the CEJ, therefore this study is important to ASESS the microleakage and internal voids in Class II restorations using different intermediate materials with the open sandwich technique.

## Results:

The present in vitro study was conducted to evaluate and compare gingival margin microleakage and internal void formation among three different intermediate restorative materials used in the open sandwich technique for Class II composite restorations. The restorative materials evaluated included resin-modified glass ionomer cement (RMGIC), ACTIVA Bioactive restorative material, SDR bulk-fill flowable composite, and a control group restored directly using nanohybrid packable composite resin without any intermediary material. The restorations were assessed under a stereomicroscope with cavity margins positioned either 1 mm coronal or 1 mm apical to the cemento-enamel junction (CEJ). As shown in Table 1, statistically significant differences in microleakage were observed among the study groups for both coronal and apical margin locations. When the gingival margins were positioned 1 mm coronal to the CEJ (Group A), the control group restored with Tetric N Ceram exhibited the highest mean microleakage score (2.8333 ± 0.75277). In contrast, ACTIVA Bioactive demonstrated the lowest microleakage score (1.1667 ± 1.32916), followed closely by RMGIC (1.3333 ± 0.5164) and SDR (1.3333 ± 1.36626). The overall intergroup comparison showed statistical significance (F = 3.284, p = 0.042), indicating differences in marginal sealing ability among the restorative approaches when the margins were located in enamel. Similarly, when the cavity margins were positioned 1 mm apical to the CEJ (Group B), significant differences were also observed among the groups, as illustrated in Table 1 (F = 6.194, p = 0.004). The highest microleakage was again observed in the control group restored solely with nanohybrid composite resin (3.6667 ± 0.5164). ACTIVA Bioactive exhibited the least microleakage (0.6667 ± 1.21106), followed by RMGIC (1.5 ± 1.3784), whereas SDR demonstrated comparatively higher leakage values (2.6667 ± 1.75119). These findings indicate that intermediary restorative materials improved marginal adaptation and reduced leakage, particularly in cavities extending below the CEJ where bonding to dentin and cementum is more challenging. Furthermore, comparison between coronal and apical margin groups revealed that restorations with margins placed apical to the CEJ demonstrated significantly greater microleakage overall. This may be attributed to the reduced bonding predictability of dentin and cementum compared to enamel, owing to their higher organic content and moisture presence. The distribution of internal voids within the restorations is presented in Table 2. Internal voids were evaluated at the interface region, cervical region, and occlusal region. ACTIVA Bioactive demonstrated the least number of voids overall, with only one interface void, no cervical voids, and one occlusal void. SDR and RMGIC also showed relatively low frequencies of void formation, while the control group exhibited the highest number of voids in all evaluated regions. At the cervical region specifically, the control group demonstrated five specimens with voids, whereas ACTIVA Bioactive showed complete absence of cervical voids. Although these differences were not statistically significant, the findings suggest superior internal adaptation and handling characteristics of ACTIVA Bioactive compared to the other materials tested. The pairwise intergroup comparisons obtained using Post-Hoc Tukey's test are summarized in Table 3. In Group A (coronal margins), no statistically significant differences were observed between the intermediary material groups and the control group, although comparisons involving the composite control group approached significance. This suggests that when the gingival margin is located in enamel, the various restorative materials perform relatively similarly with respect to marginal sealing. In contrast, for Group B (apical margins), significant differences were observed between ACTIVA Bioactive and the composite control group (p = 0.003), as well as between RMGIC and the composite control group (p = 0.041), as shown in Table 3. These findings indicate that the use of ACTIVA Bioactive and RMGIC significantly reduced microleakage compared to direct composite restorations when margins extended below the CEJ. No statistically significant differences were observed between SDR and the other intermediary materials. Overall, the findings of the present study indicate that the use of intermediary restorative materials in the open sandwich technique can reduce gingival microleakage and internal void formation, especially in restorations with cervical margins extending apical to the CEJ. Among all the materials evaluated, ACTIVA Bioactive demonstrated the most favorable performance with the least microleakage and minimal internal void formation, suggesting superior marginal adaptation and sealing ability.

## Discussion:

This study evaluated microleakage and internal void formation associated with different intermediate materials placed beneath composite restorations in Class II cavities, with margins located both coronal and apical to the cementoenamel junction (CEJ). Microleakage remains a critical concern in restorative dentistry, primarily attributed to polymerization shrinkage during composite curing, which generates stresses at the tooth-restoration interface and leads to marginal gap formation. This phenomenon is strongly associated with post-operative sensitivity and secondary caries, ultimately contributing to restoration failure. Gingival margins positioned in dentin or cementum are particularly vulnerable due to the absence of enamel, which otherwise provides a more favorable substrate for adhesion because of its higher inorganic content [14]. In this context, Ouzel *et al.* (2008) [15] reported that margins located coronal to the CEJ demonstrated significantly lower microleakage compared to those placed apically. Internal voids constitute another significant limitation of composite restorations, adversely affecting mechanical properties such as tensile strength, abrasion resistance, and water sorption and color stability. These voids act as stress concentrators, increasing the likelihood of fracture propagation and restoration failure under functional loading. To mitigate both microleakage and void formation, the sandwich technique employing an intermediate material with a low modulus of elasticity has been advocated to absorb polymerization stresses and enhance marginal adaptation [16]. In the present study, thermocycling was employed to simulate intraoral conditions and aging, thereby enhancing the clinical relevance of the findings. Dye penetration using rhodamine B was utilized to assess marginal integrity due to its superior penetration capability and small molecular size, enabling detection of even minimal interfacial gaps. The results revealed that ACTIVA Bioactive exhibited the least microleakage, while the nanohybrid packable composite control group demonstrated the highest. RMGIC and SDR showed comparable performance when margins were placed coronal to the CEJ; however, in apical margins, RMGIC performed slightly better, closely followed by SDR [17]. With respect to internal voids, ACTIVA Bioactive demonstrated the lowest incidence, followed by SDR and RMGIC, whereas the control group exhibited the highest number of voids. These findings are consistent with Samet *et al.* (2006) [18], who attributed increased void formation in packable composites to their higher stiffness, which limits proper adaptation to cavity walls. Similarly, Lokhande *et al.* [19] reported that flow able composites effectively reduce interfacial voids, a finding corroborated in this study with SDR, likely due to its superior flow ability, self-leveling behavior and low modulus of elasticity,which collectively enhance adaptation and reduce polymerization stress. RMGIC demonstrated improved marginal sealing due to its dual chemical and mechanical bonding capabilities, as noted by previous studies; however, it showed comparatively higher void formation in deep cavities, likely due to its lower flow and technique sensitivity. In contrast, ACTIVA Bioactive consistently outperformed other materials in minimizing both microleakage and voids. Ghilotti *et al.* (2023) [20] reported similar findings, attributing its superior performance to its bioactive ionic resin matrix, which facilitates chemical interaction with tooth structure and promotes the formation of a stable resin-hydroxyapatite complex. Overall, microleakage is influenced by multiple factors, including polymerization shrinkage, thermal stresses and material compatibility. While techniques such as incremental layering and C-factor control have been proposed, the use of an appropriate intermediate material appears to significantly enhance restoration integrity. However, further long-term clinical studies are necessary to validate these findings and establish the durability of bioactive materials like ACTIVA Bioactive in routine practice.

## Conclusion:

ACTIVA Bioactive showed superior performance in minimizing microleakage at both coronal and apical margins, followed by RMGIC and SDR. RMGIC and SDR showed comparable results at coronal margins, while RMGIC performed slightly better than SDR at apical margins. In terms of internal voids, ACTIVA Bioactive exhibited the least void formation, followed by SDR and RMGIC.

## Figures and Tables

**Table 1:** Microleakage

**Groups**		**Mean**	**Std. Deviation**	**F-value**	**P-value**
A	Tetric N Ceram	2.8333	0.75277	3.284	0.042
	RMGIC	1.3333	0.5164		
	ACTIVA Bioactive	1.1667	1.32916		
	SDR	1.3333	1.36626		
B	Tetric N Ceram	3.6667	0.5164	6.194	0.004
	RMGIC	1.5	1.3784		
	ACTIVA Bioactive	0.6667	1.21106		
	SDR	2.6667	1.75119		

**Table 2:** Internal voids

**Groups**	**Interface void**		**Cervical void**		**Occlusal void**	
	0	1	0	1	0	1
Tetric N Ceram -	27	3	25	5	26	4
RMGIC	27	3	28	2	27	3
ACTIVA Bioactive	29	1	30	0	29	1
SDR	28	2	29	1	28	2

**Table 3:** Post Hoc Tukey's test results for comparison of microleakage and internal voids

**Dependent Variable**	**(I) gp**	**(J) gp**	**(I-J)**	**Sig.**
A	RMGIC	RMGIC	-0.16667	0.993
		SDR	-0.16667	0.993
		Composite	-1.66667	0.057
	ACTIVA	Composite	0	1
	Bioactive	SDR	-1.5	0.098
	SDR	Composite	-1.5	0.098
B	ACTIVA	RMGIC	-0.83333	0.685
	Bioactive	SDR	-2	0.064
		Composite	-3.00000*	0.003
	RMGIC	SDR	-1.16667	0.422
		Composite	-2.16667*	0.041
	SDR	Composite	-1	0.55
